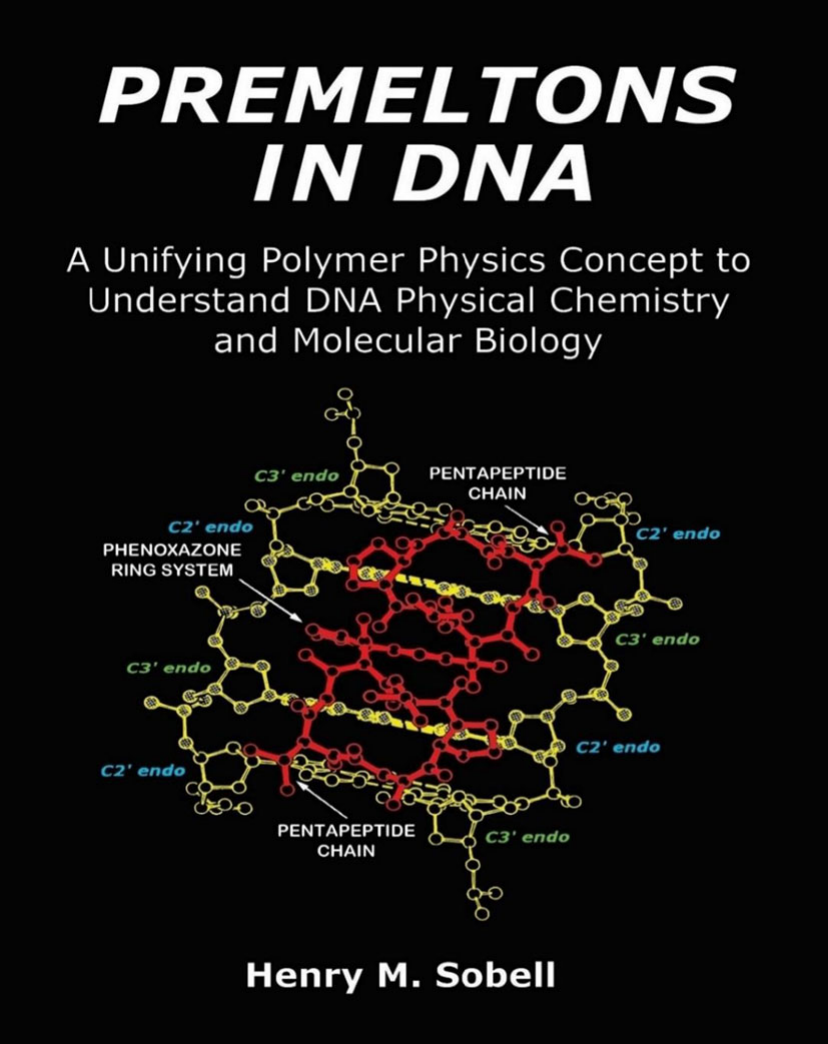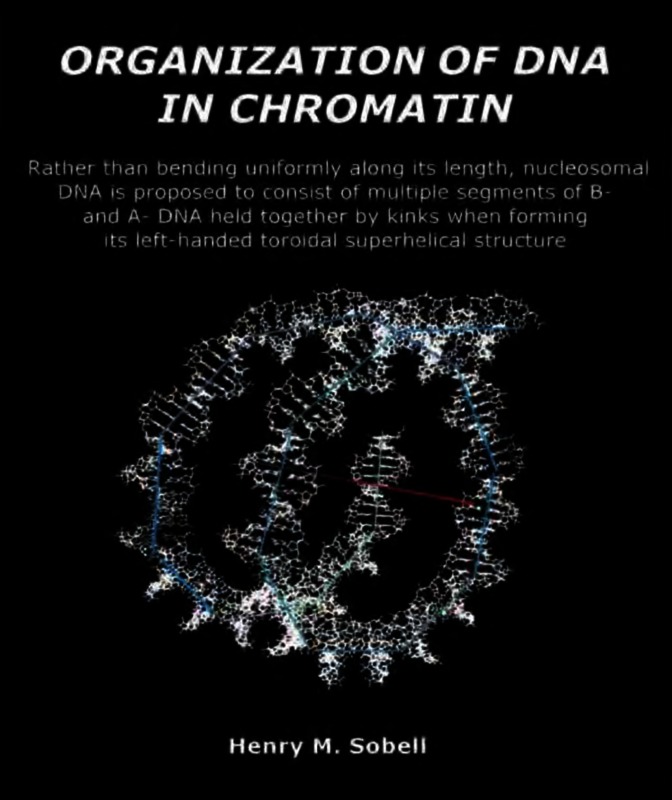# Premeltons in DNA

**DOI:** 10.1007/s10969-016-9202-4

**Published:** 2016-03-16

**Authors:** Henry M. Sobell

**Affiliations:** Departments of Chemistry and Molecular Biophysics, University of Rochester, Rochester, NY 14642 USA

**Keywords:** Premeltons, Meltons, Intercalation, Ethidium-DNA binding, Actinomycin-DNA binding, B- to A- DNA structural phase-transition, DNA-melting and premelting, Gene-regulation, RNA-polymerase: promoter recognition, Protein: DNA allosterism, DNA transcription, Initiation, Elongation, Termination of RNA-synthesis

## Abstract

Premeltons are examples of emergent-structures (i.e., structural-solitons) that arise spontaneously in DNA due to the presence of nonlinear-excitations in its structure. They are of two kinds: B–B (or A–A) premeltons form at specific DNA-regions to nucleate site-specific DNA melting. These are stationary and, being globally-nontopological, undergo breather-motions that allow drugs and dyes to intercalate into DNA. B–A (or A–B) premeltons, on the other hand, are mobile, and being globally-topological, act as phase-boundaries transforming B- into A-DNA during the structural phase-transition. They are not expected to undergo breather motions. A key feature of both types of premeltons is the presence of an intermediate structural-form in their central regions (proposed as being a transition-state intermediate in DNA-melting and in the B- to A-transition), which differs from either A- or B-DNA. Called beta-DNA, this is both metastable and hyperflexible—and contains an alternating sugar-puckering pattern along the polymer backbone combined with the partial unstacking (in its lower energy-forms) of every-other base-pair. Beta-DNA is connected to either B- or to A-DNA on either side by boundaries possessing a gradation of nonlinear structural-change, these being called the kink and the antikink regions. The presence of premeltons in DNA leads to a unifying theory to understand much of DNA physical chemistry and molecular biology. In particular, premeltons are predicted to define the 5′ and 3′ ends of genes in naked-DNA and DNA in active-chromatin, this having important implications for understanding physical aspects of the initiation, elongation and termination of RNA-synthesis during transcription. For these and other reasons, the model will be of broader interest to the general-audience working in these areas. The model explains a wide variety of data, and carries with it a number of experimental predictions—all readily testable—as will be described in this review.


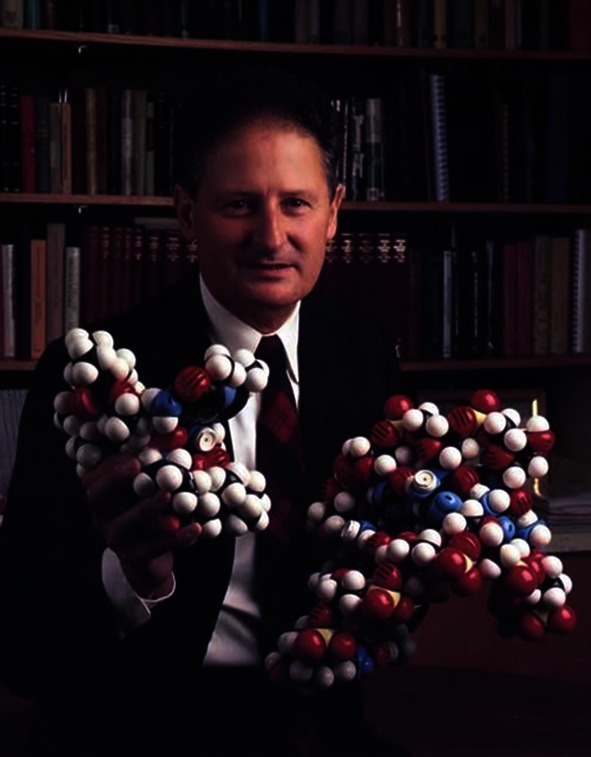
This photo—taken a number of years ago—shows me holding CPK space filling molecular models of actinomycin D intercalating into (what I have called) the beta-DNA structure, this being a metastable and hyperflexible liquid‐like phase that acts as a transition-state intermediate in DNA melting.

Beta-DNA is proposed to arise within entities called premeltons—these having dynamic structural properties that allow drugs and dyes to intercalate into its structure. Their presence in DNA leads to a unifying conceptual theory to understand much of DNA physical-chemistry and molecular-biology.

In particular, premeltons are predicted to arise within the early melting-regions in DNA—many of these defining the 5′ and 3′ ends of genes in both naked DNA and DNA in active-chromatin. Their presence at the beginning and ends of genes has important repercussions for understanding physical aspects of the initiation, elongation and termination of RNA-synthesis during DNA transcription.

For these and other reasons, the model will be of broader interest to the general audience working in these areas. It makes a number of key experimental predictions—all readily testable—as will be pointed out in this review.

We begin by reviewing evidence that indicates beta-DNA to be a key metastable and hyperflexible liquid-like phase—whose presence in DNA allows drugs and dyes to intercalate into its structure.

After our initial studies with actinomycin [1–5], we continued to discover a large number of additional crystalline complexes containing the planar-intercalators shown in Fig. 1, complexed to a series of self-complementary DNA- and RNA- like dinucleoside-monophosphates. Their interactions with these nucleic-acid fragments are simple, meaning that they exclusively utilize stacking interactions with the base-pairs and electrostatic interactions with the sugar-phosphate chains to stabilize their structures.

**Fig. 1:**
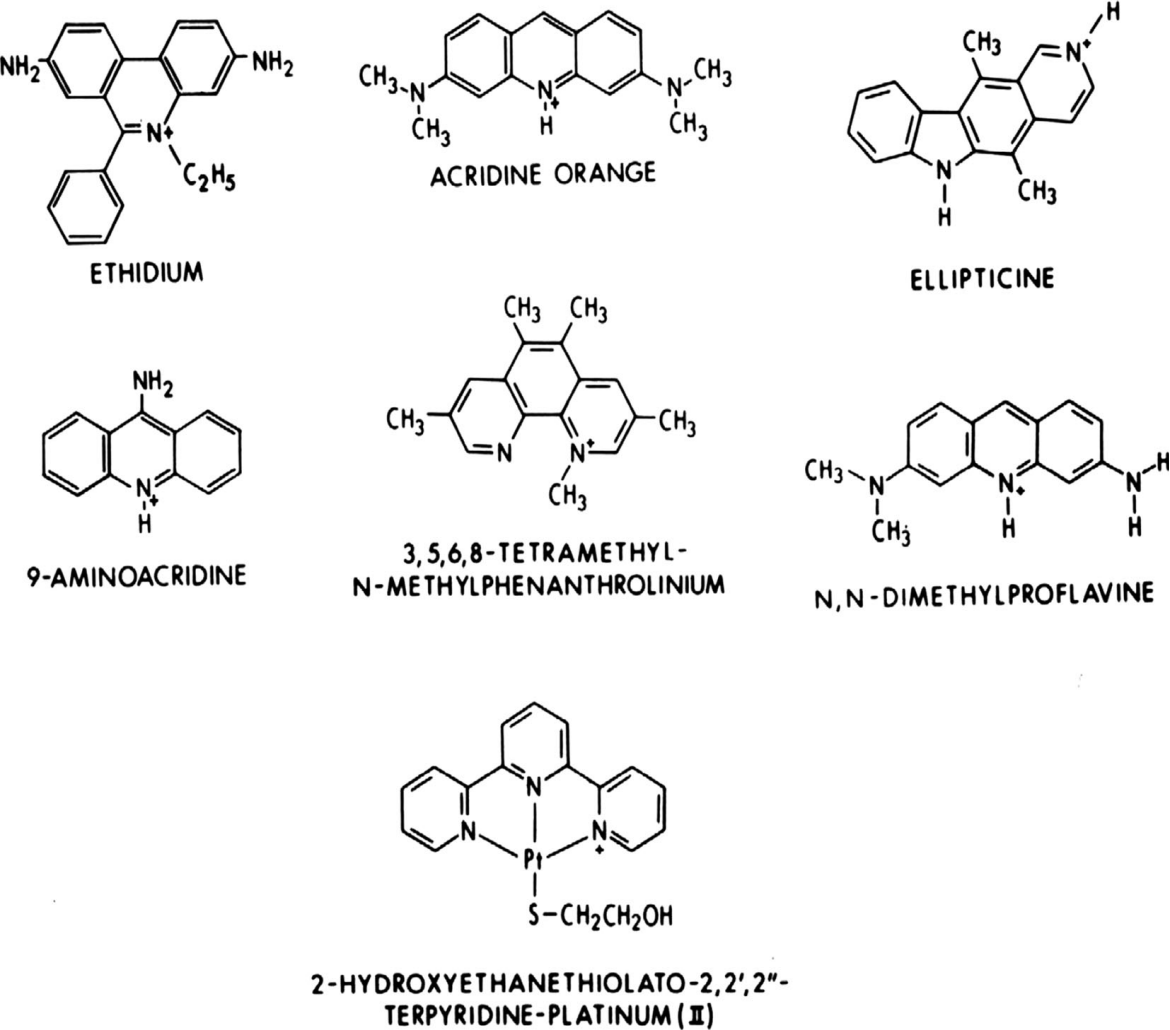
Chemical structures of simple intercalators

One such structure contains ethidium complexed to ribo-CpG [18]—this is shown in Fig. 2.

**Fig. 2:**
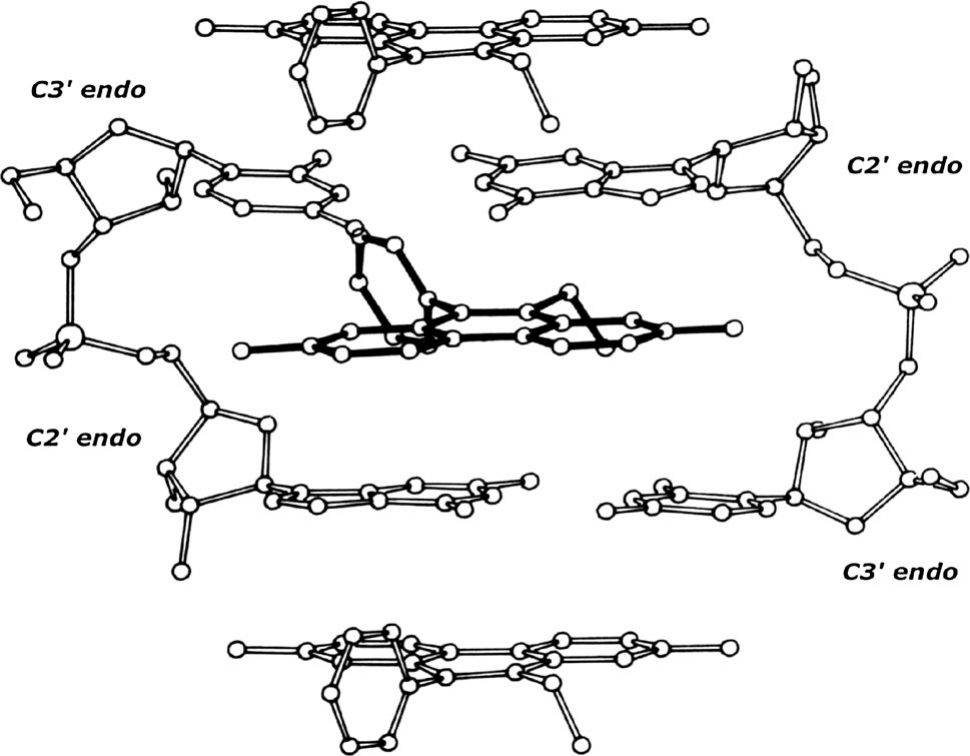
The structure of a 2:2 ethidium: ribo-CpG crystalline-complex

The complex consists of an intercalated ethidium-molecule (shown with dark covalent bonds), and stacked ethidium-molecules (shown with light covalent bonds) located above and below the intercalated complex.

Sugar-phosphate chains demonstrate the mixed sugar-puckering pattern: [C3′ *endo* (3′–5′) C2′ *endo*]—which allow base-pairs to separate 6.7 Angstroms, and to remain twisted relative to one another by about 10°.

We refer to this base‐paired dinucleoside-monophosphate complex as being the highest-energy form of the beta-structural element—“pinned” by ethidium.

This beta-structural element has been observed in 15 separate crystallographic determinations. These involve seven different intercalators complexed to a variety of DNA-like and RNA-like dinucleoside monophosphates. Four structures are isomorphous and, therefore, demonstrate a host–guest relationship. The remaining eleven structures crystallize in different lattice environments that contain varying numbers of water molecules [6–20].

This structural information readily leads to the ethidium-DNA neighbor-exclusion binding-model shown in Fig. 3 [21–29].

**Fig. 3:**
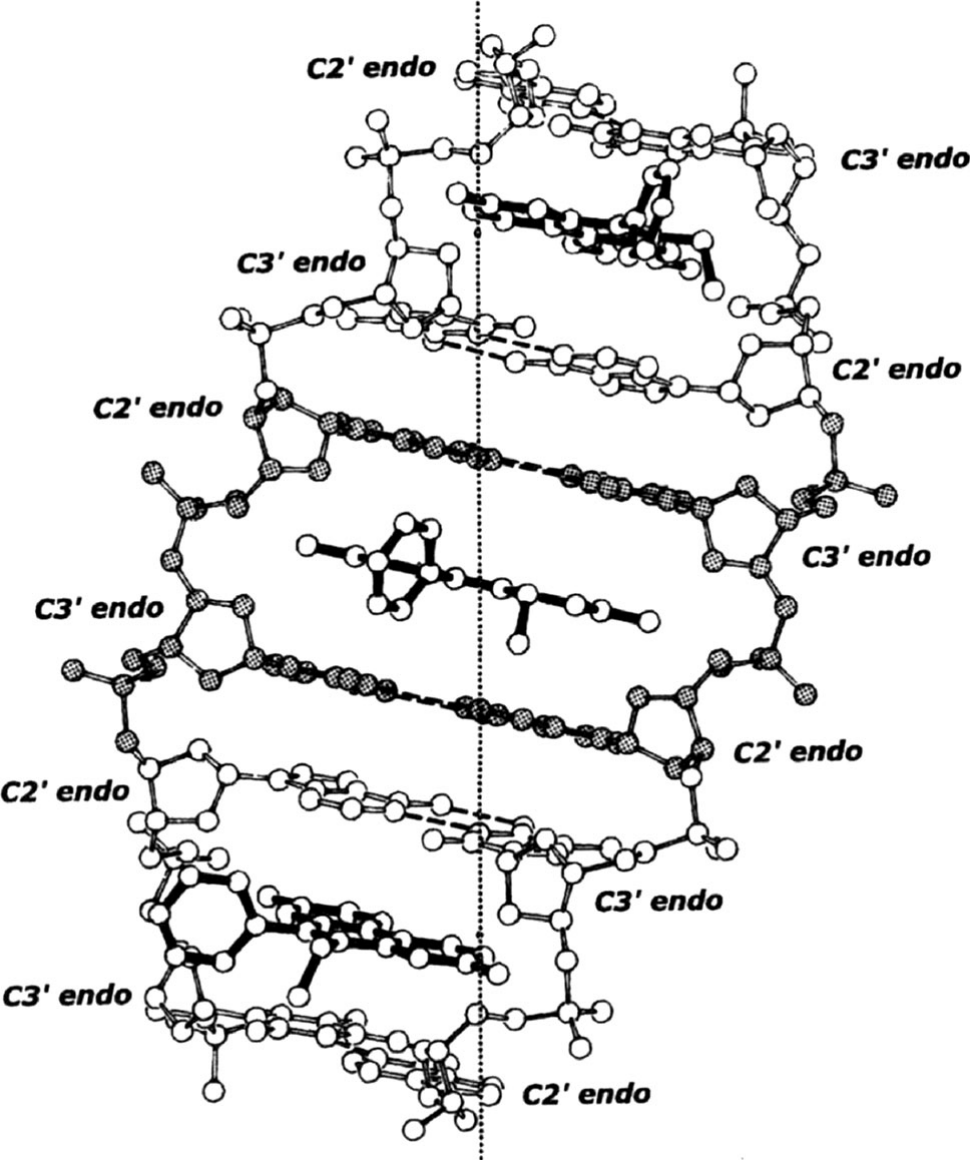
The ethidium-DNA neighbor-exclusion binding-model

The beta-structural element plus ethidium form the asymmetric-unit of the helix—a repeated twist of 47.2°, and a translation of 9.8 Angstroms along the helix-axis—generates the helical complex shown.

*It should be noted that intercalation occurs between every-other base-pair, since binding is restricted to neighboring beta‐structural elements*. This feature explains the magnitude of DNA stretching and unwinding accompanying neighbor-exclusion binding.

Notice that the stereochemistry connecting neighboring beta-structural elements is different [i.e., C2′ *endo* (3′–5′) C3′ *endo*]. There is no significant stretching or unwinding in this region.

An important prediction of this neighbor-exclusion binding model is that extended microcrystalline domains form at high-drug/DNA binding ratios. This prediction has been confirmed by fiber-diffraction studies (shown in Fig. 4) [28], which indicate the platinum organometallo-intercalator [2-hydroxyethane-thiolato (2, 2′,2″ terpyridine) platinum (II)] to form extended microcrystalline domains when it complexes with calf-thymus DNA at high-drug/DNA binding ratios.

**Fig. 4:**
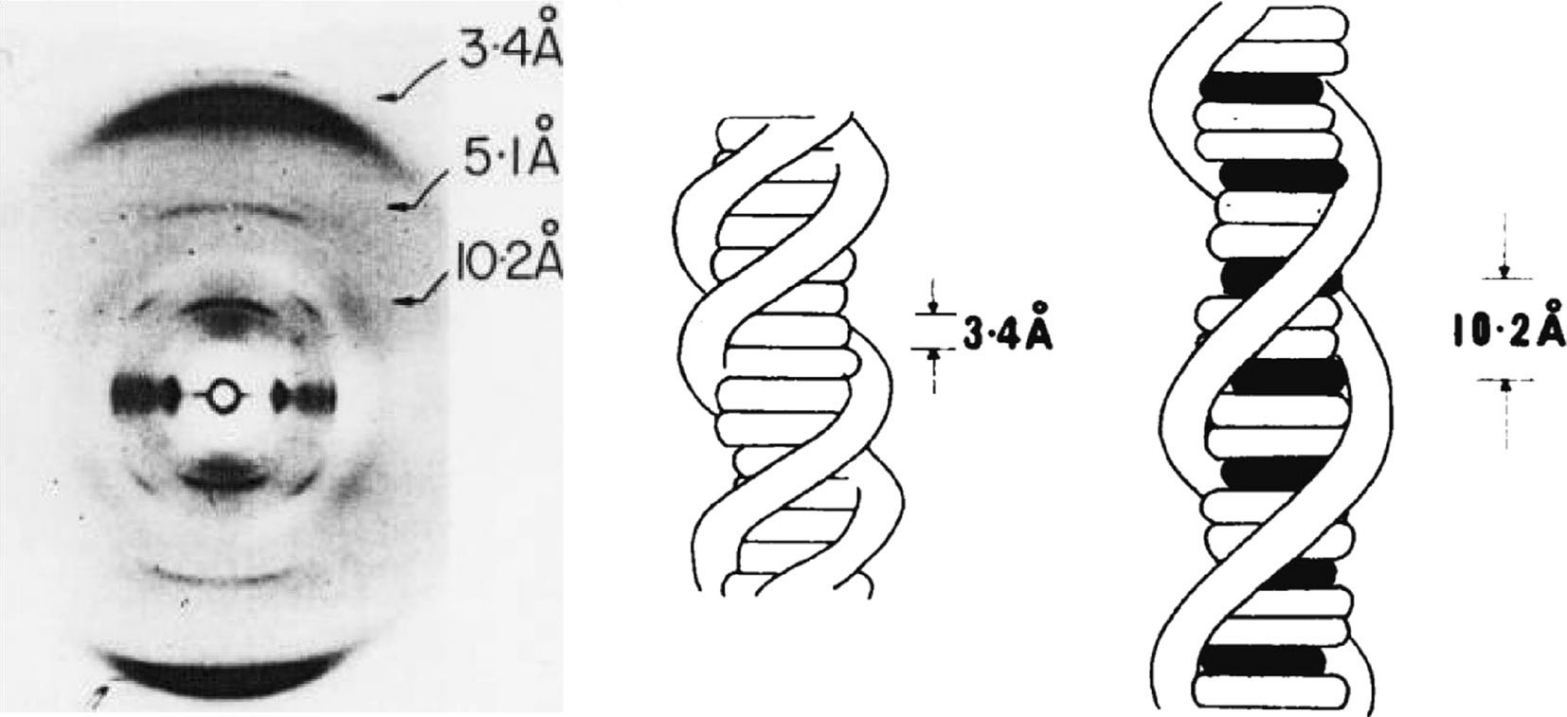
An X-ray fiber diffraction-pattern obtained from polycrystalline-fibers containing 2-hydroxyethane-thiolato (2, 2′, 2″ terpyridine) platinum (II) bound to calf-thymus DNA. This study confirms the presence of neighbor-exclusion binding by this platinum organometallo-intercalator upon binding to DNA at high drug/DNA ratios [28]

From the sharpness of the 10.2 and 5.1 Angstrom near meridianal reflections (these primarily reflecting the platinum–platinum scattering vectors), the crystalline domain size in these fibers can be estimated to be in the order of several hundred (intercalated) base-pairs [30]—*this most easily being understood as arising from a structural phase transition*—*in which* the *platinum organo-metallointercalator: highest-energy beta-DNA complex arises as its dominant phase*.

As shown in Fig. 5, the beta-DNA structure is expected to be both *metastable* and *hyperflexible*, and therefore—to exist in many different energy states. It is bounded on the left by its lowest energy state, and on the right by its highest-energy state.

**Fig. 5:**
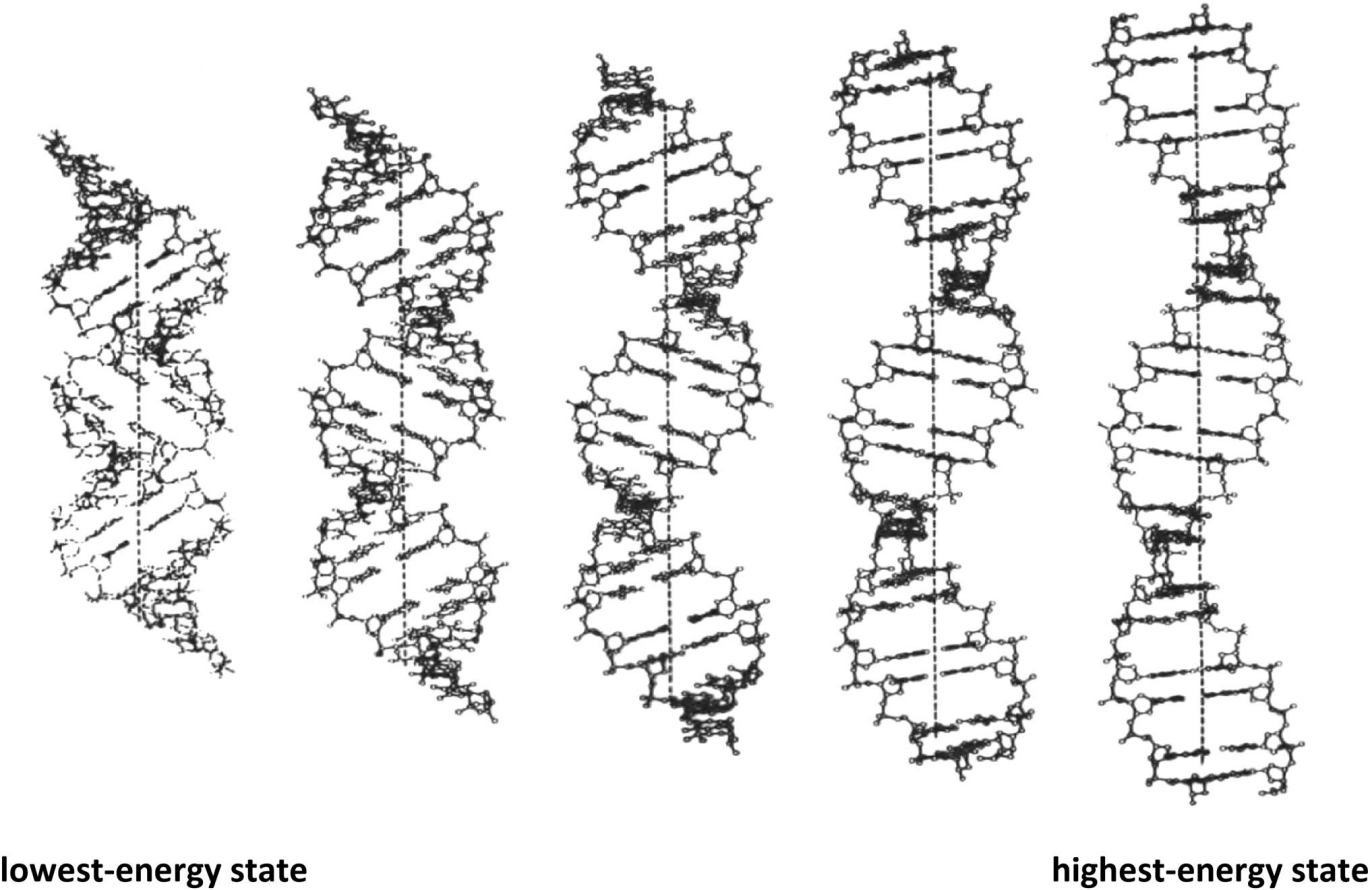
Beta-DNA is proposed to be both *metastable* and *hyperflexible*, and therefore to exist in many different energy states. It is bounded on the left by its lowest-energy state, and on the right by its highest-energy state. For comparative purposes, each structure contains 20 base-pairs

Steroidal-diamines such as irehdiamine A [31–34] stabilize its lowest-energy state by partial intercalation, while planar drugs and dyes, such as ethidium [32, 35]—stabilize its highest-energy state by complete intercalation.

The lowest-energy state is proposed to be a transition-state intermediate in the B- to A transition, while its highest‐energy state—being a maximally extended and unwound DNA duplex structure—is proposed to be a transition-state intermediate in DNA melting.

**Fig. 6:**
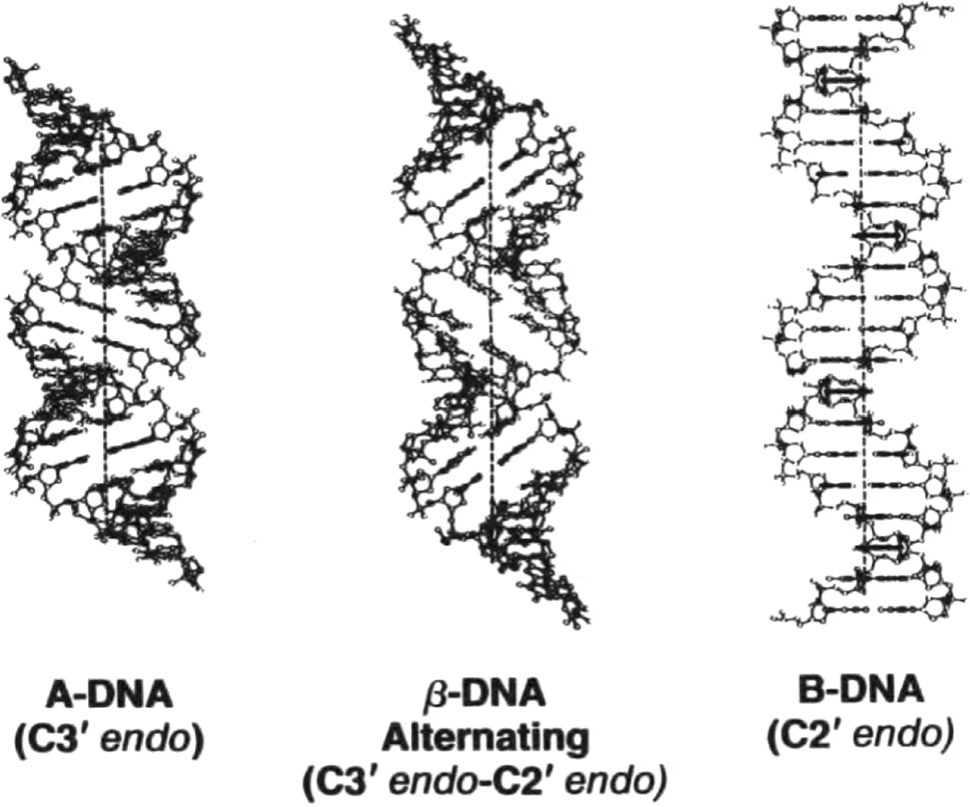
A-DNA, beta-DNA and B-DNA, and their associated sugar-pucker conformations. For comparative purposes each structure shown contains 20 base-pairs

The lowest-energy beta-DNA form (shown in Fig. 6) has helical-parameters midway between those of A- and B-DNA—suggesting it to be a *transition-state intermediate in the B- to A-transition*.
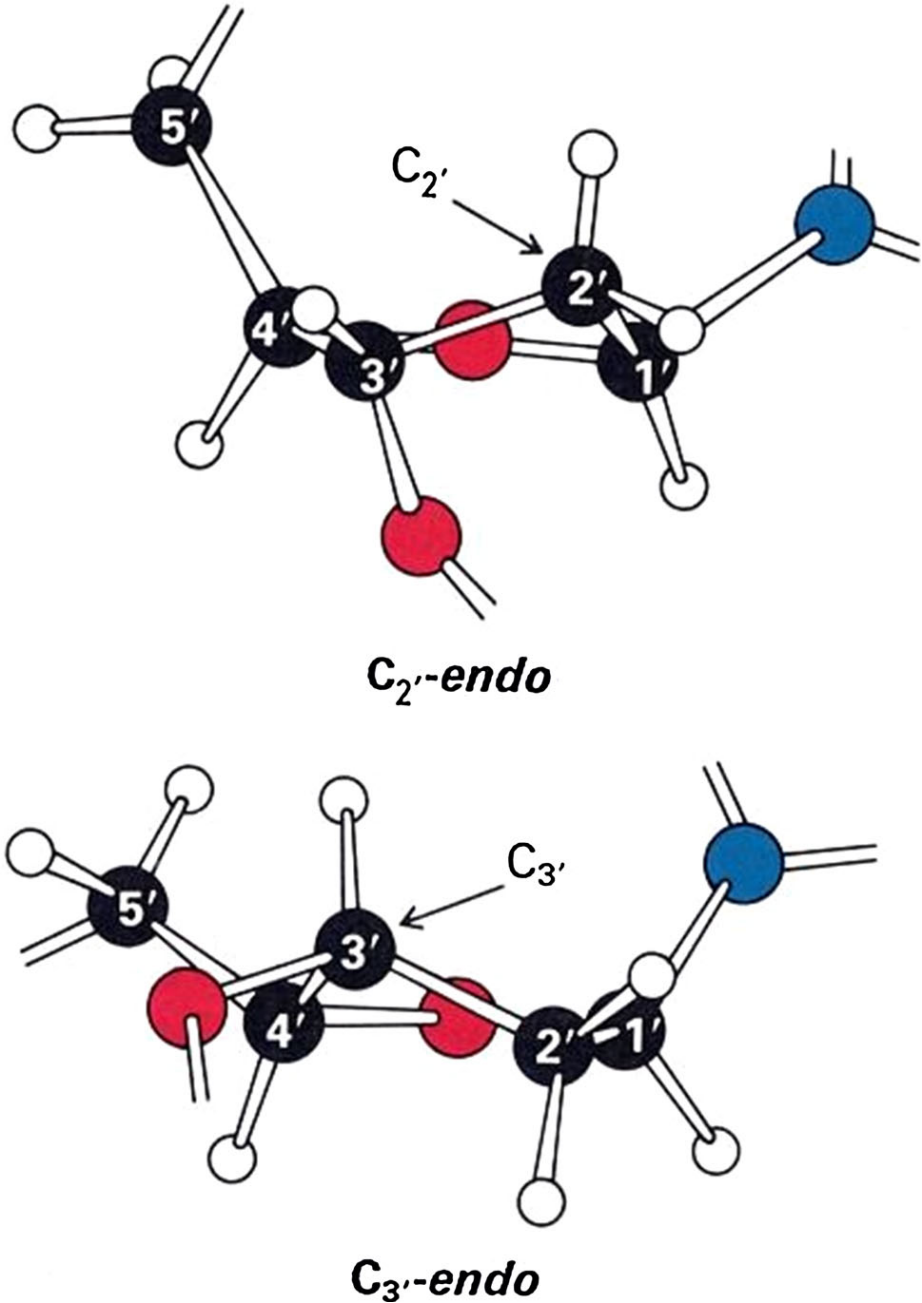


*Note*: Deoxyribose sugar-residues, both as individual molecules or joined within the polymer-structure, can assume either *C2′ endo* or *C3′ endo* pucker-conformations, both conformations having similar energies. Through the use of the pseudo-rotational-parameter (a mathematical parameter that defines the sugar-conformation), one can explore the energies of the complete range of conformational states. These calculations show energy minima at *C2′* and *C3′ endo* regions, these being connected by a minimal-energy pathway having a barrier of about 1.5 kcal/mole. In B-DNA, sugar-residues have *C2′ endo* puckers, whereas in A-DNA, they have *C3′ endo* puckers. The transition-region separating these two sugar-pucker conformations is, therefore, a key source of nonlinearity that separates the A- and B- conformational states. Beta-DNA utilizes a similar source of nonlinearity i.e., the beta structural element contains *bothC3′ endo* and *C2′ endo* sugar-puckers [i.e., *C3′ endo* (3′–5′) *C2′ endo*] to distinguish it from the A- and B-forms. Its metastability reflects the presence of additional energies in its structure that necessitate the partial-unstacking of alternate base-pairs (i.e., within each beta-structural element) in its lowest-energy form.

Using the technique of linked-atom least squares [36], it has been possible to compute structural intermediates that lie along the minimal-energy pathway connecting B- with A-DNA—refer to Fig. 7a, b. This has been accomplished by calculating a series of uniform-transitions along the polymer, in which the puckering of every *other* deoxyribose-sugar was altered incrementally, and the structures then energy-minimized subject to series of constraints and restraints. In this way, we have discovered the existence of a minimal-energy pathway connecting B- with A- DNA, which passes through its lowest-energy beta‐DNA form.

**Fig. 7:**
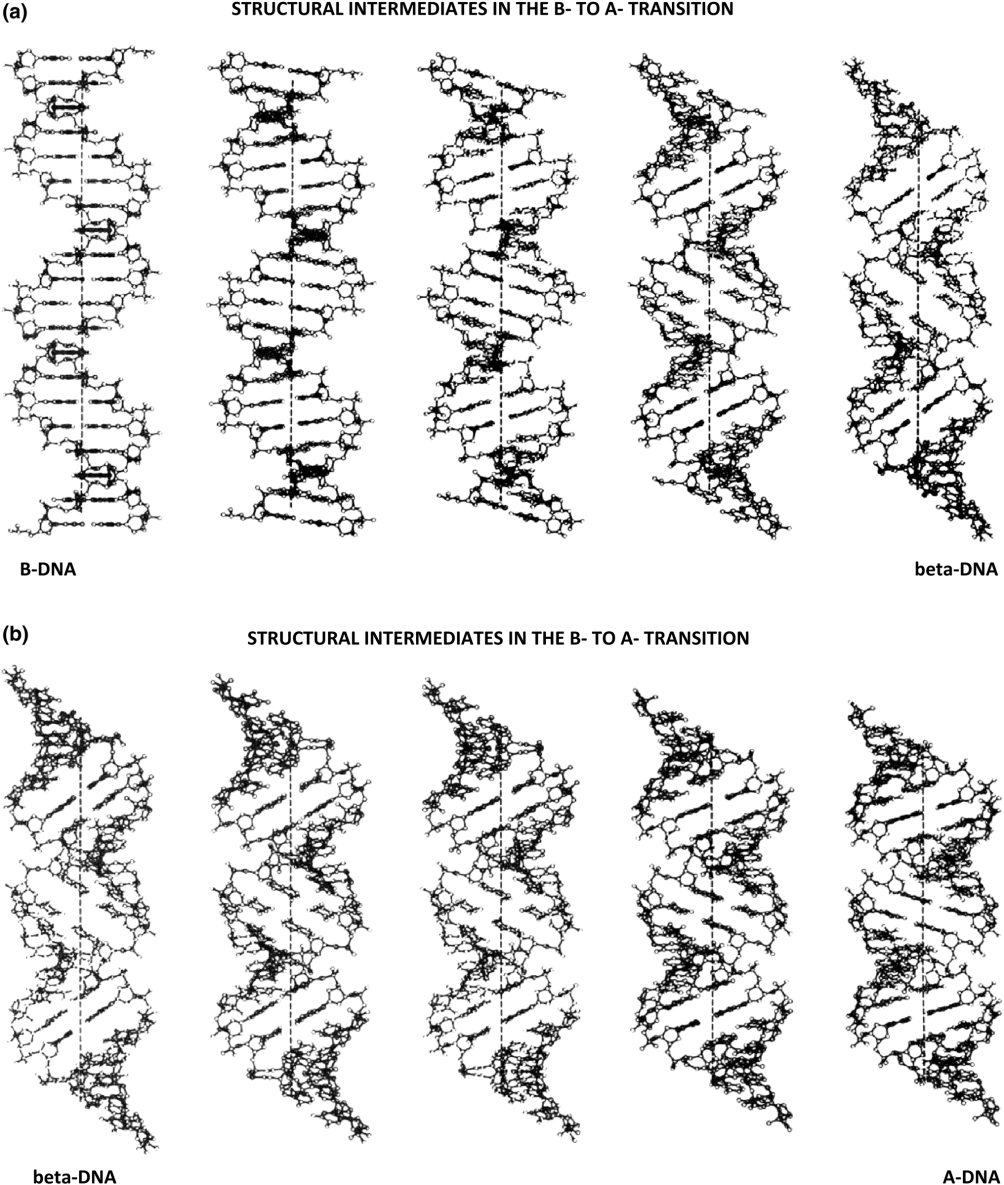
**a**, **b** Structural intermediates connecting B-DNA with A-DNA, computed as a uniform-transition along the polymer-chain by the method of linked-atom least-squares [36]. For comparative purposes, each figure contains 20 base-pairs

*Note*: Detailed calculations have shown that there is little or no base-pair unstacking in the first-half of the B- to beta-DNA (and the A- to beta-DNA) structural intermediates [37]. The combination of DNA-unwinding, counterbalanced by right-handed superhelical writhing, is achieved almost entirely, by “rolling” adjacent base-pairs (upon each-others van der Waals surfaces) towards the wide-groove direction, accompanied by a gradual modification of (alternate) sugar-pucker geometries within the polymer-backbone. As one passes over the energy-barrier separating *C2′ endo* from *C3′ endo* sugar-conformations, there is a more abrupt-onset of partial base-pair unstacking to relieve the strain-energies in the sugar-phosphate chain that would otherwise-develop. We have found it necessary to relax the exact-requirement that only alternate-sugars are involved in the transition. To get over the energy-barriers arising in these intermediate states, it is necessary to gently “rock” the other sugar-residues “backwards”, toward the *C2′ exo* conformation (in the B- to beta-pathway), or towards the *C3′ exo* conformation (in the A- to beta- pathway)—this readily allows passage through these barriers.

Twenty-five structural intermediates have been calculated by this procedure—although for simplification, only nine have been shown here. In these calculations, physicists will recognize sugar‐puckers to be the “masters”, torsional angles defining the sugar-phosphate and base-sugar conformations, the “slaves” [38–40]. Final coordinates for all twenty-five structural intermediates—along with the ethidium-DNA neighbor-exclusion binding-model—have been published [37].

These calculations were followed by a least-squares procedure, in which adjacent dinucleotide elements from each structure were linked together to form the *two different kinds* of premeltons shown in Fig. 8.

**Fig. 8:**
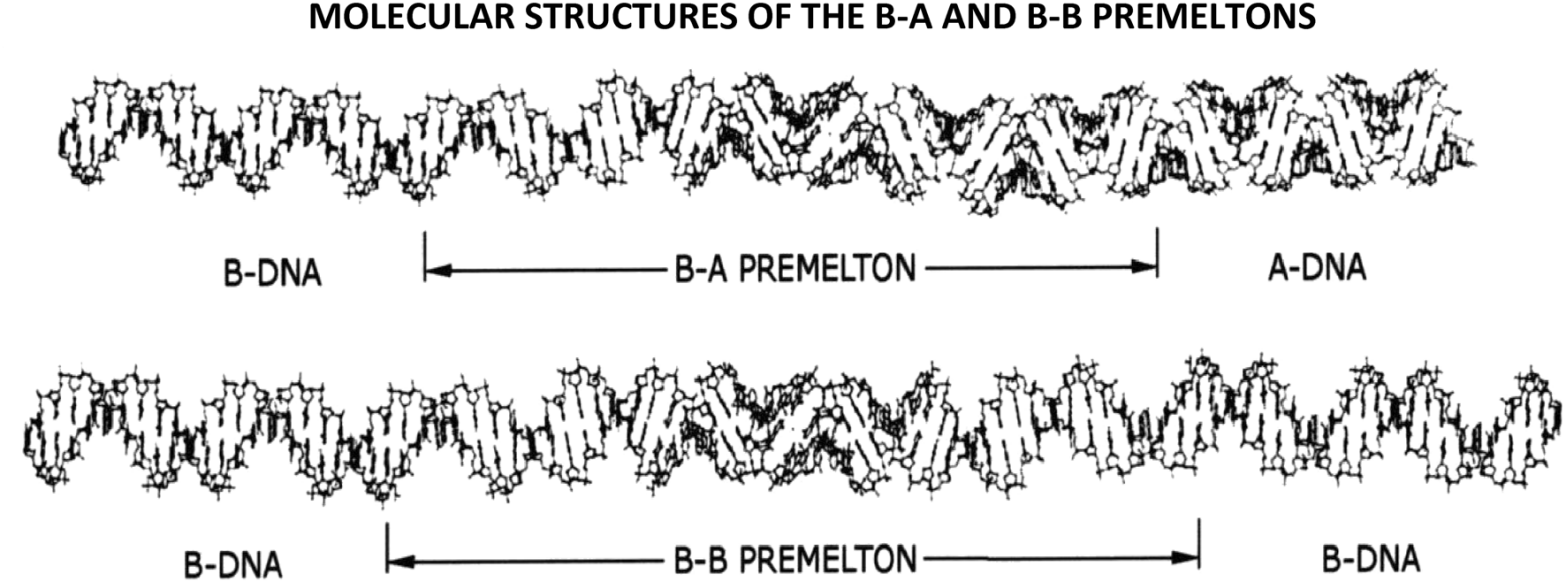
The molecular-structures of B–A and B–B premeltons. To simulate these structures, base-paired dinucleotide-elements obtained from the modeling-studies described in Fig. 7a, b, were pieced-together using a least-squares procedure. It is seen that, whereas B-A premeltons are (globally) *topological*, B–B premeltons are (globally) *nontopological*—*this reflects the presence of a bifurcation*—which gives rise to these two different-types of structural-solitons in DNA. See text for further discussion

It is seen that, whereas B-A (or A-B) premeltons are (globally) topological, B–B (or A–A) premeltons are (globally) nontopological—this reflecting the presence of a bifurcation, which gives rise to these two different-types of structural-solitons in DNA.

*Note*: A bifurcation is defined as an event that takes place at a branch-point in a pathway to give rise to two different outcomes. Although the source of the nonlinearity (that determines the pathway) remains the same, the decision to which pathway to take at the branch point is influenced by a bias. In the case of the B- to A-transition originating within the centers of premeltons, prevailing thermodynamic-conditions provide the bias.

B–B (or A–A) premeltons form at specific DNA regions to nucleate site-specific DNA melting. They are stationary and – being (*globally*) *nontopological*—are predicted to undergo breather-motions that allow drugs and dyes to intercalate into DNA.

B–A (or A–B) premeltons, on the other hand, are mobile—and, being (*globally*) *topological*—act as phase-boundaries transforming B- into A-DNA during the structural phase-transition. They are not predicted to undergo breather-motions.

More generally, phase-boundaries connecting the central beta-DNA region with either B- or A-DNA on either side—are referred to by physicists and mathematicians as the “kink” and the “antikink”—the premelton being an example of a “kink-antikink bound-state”. The modulated beta‐alternation in sugar-puckering, in combination with the partial-unstacking of alternate base-pairs within these kink and antikink boundaries, reflects the presence of the *Peierl’s distortion* [41]—a spontaneous dimerization known to occur within solitons that arise in other polymers (i.e., the *polaron* in *trans*-polyacetylene—an electronic-soliton that gives rise to its superconductive properties [42]).

*Note*: The terms “kink” and “antikink” have been used by both physicists and applied-mathematicians to describe the solutions to a large-number of nonlinear partial differential-equations—they have precise meaning, being known as “topological-solitons”. The “kink-antikink bound-state” on the other hand, represents a different class of solutions, these describing the emergence of coherent-structures that contain internal dynamical-motion (hence, the term, “breather-solitons”, or, in lattice situations, “discrete-breathers”). Kink- antikink bound-states are encountered in a large number of diverse areas in nonlinear-science, and are of particular interest to physicists and mathematicians working in these areas (readers unfamiliar with this area should consult references [38–40]). For the molecular-biologist, the word “kink” has come to mean a sharp-bend in DNA due to a highly-localized conformational-change in a sugar-residue and/or a phosphodiester-linkage [43]. Although this terminology is somewhat restricted, it has proven useful in the DNA-area and poses no problem provided physicists and biologists agree on the meaning of the word “kink” in these two different contexts.

## What are breather-motions?

**Fig. 9:**
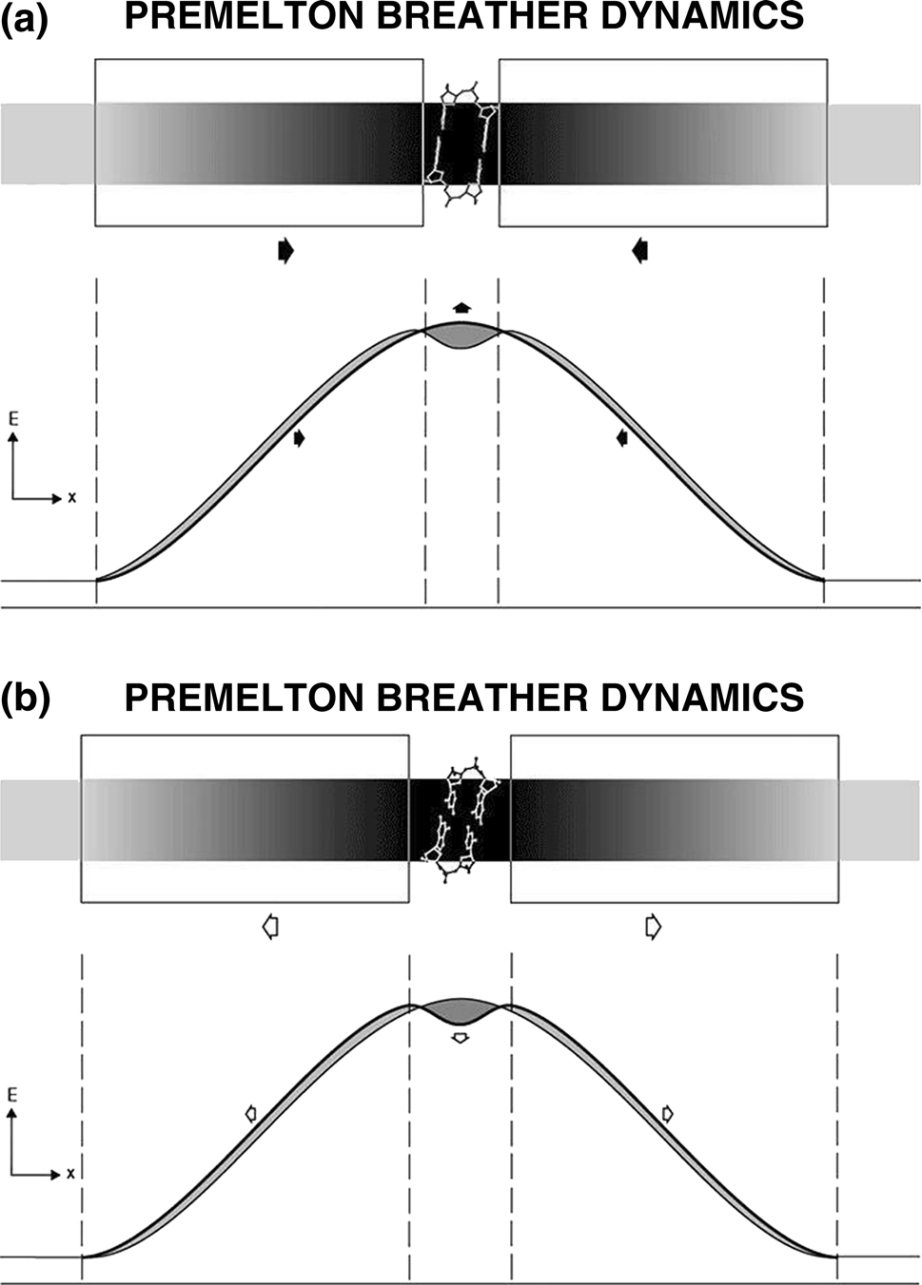
**a**, **b** Lowest-amplitude breather-motion present within a B–B (or A–A) premelton, showing its central beta-structural element alternating between its highest- and lowest-energy states

Figure 9a, b demonstrate the lowest-amplitude breather-motions present within B–B or A–A premeltons—in which the central beta-structural element alternates between its lowest- and highest-energy conformational states. These hinge-like motions are coupled with the concerted movement of the kink and antikink boundaries (shown in the boxed regions) on either side. Such boundaries act as energy domain-walls, capable of moving in and out with minimal energy-dissipation.

Note that movement in the kink- and antikink- boundaries within premeltons is tightly-coupled to the appearance of the lowest- and highest-energy states in its central beta-structural-element. The extremes of these two different energy-states, therefore, limit the excursions of the kink and antikink, causing them to remain together as a dynamical “kink-antikink bound-state”.

Isoenergetic breather-motions such as these demonstrate the collective effect, an effect well known in many areas of physics. Small movements of atoms in sugar-residues within the kink and antikink boundaries combine together to give larger movements of atoms in the central beta‐DNA core region. This collective effect explains how energy is transiently focused into the centers of premeltons to create an “open state” into which drugs and dyes intercalate.

**Fig. 10:**
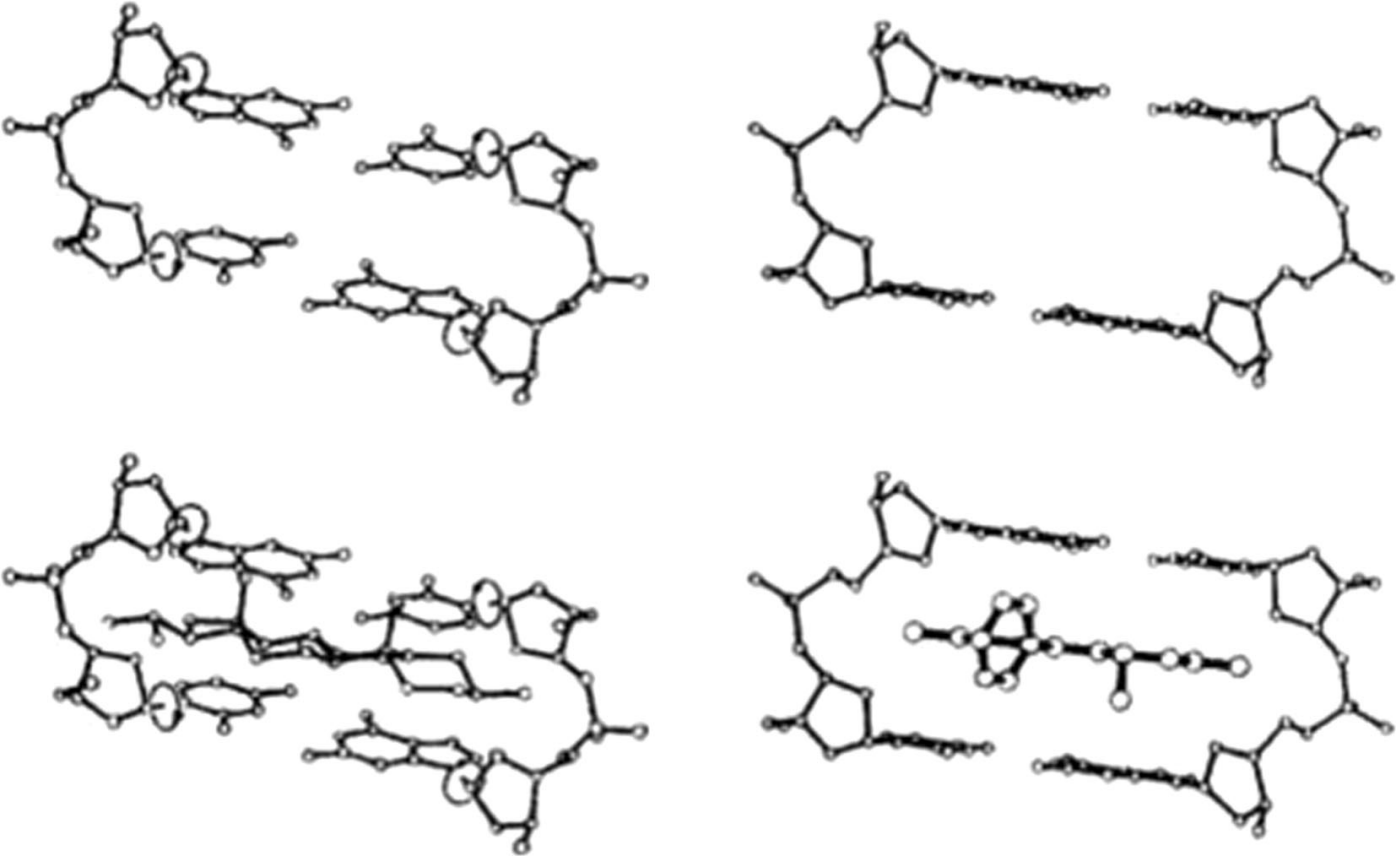
The beta-structural element—alternating between its lowest- and highest-energy states within the centers of B–B (or A–A) premeltons—“pinned” and “unpinned” by irehdiamine (*left*) and ethidium (*right*). These motions reflect the presence of dynamical breather-motions within premeltons, which facilitate the ability of drugs and dyes to intercalate into DNA

*Note*: More generally, premeltons (i.e., either of the B–B or A–A types) have been proposed to arise spontaneously within the early melting-regions of DNA (or RNA) to nucleate site-specific melting. Their presence explains the origin of pancreatic DNase I and micrococcal-nuclease hypersensitive-sites at the 5′ and 3′ ends of genes in both naked and DNA in transcriptionally-active chromatin. Since central beta-structural elements within these premeltons alternate between their lowest- and highest-energy states, they are able to act as substrates for both enzymes—pancreatic-DNase I cleaving its lowest-energy state, micrococcal-nuclease cleaving its highest-energy state. In addition, both enzymes have been shown to be capable of cleaving beta-structural elements statically present in both nucleosomal DNA, and in linker-regions connecting nucleosomes in the higher-order solenoidal-structure of chromatin. In these experiments, the chemical-nuclease, 1, 10-phenanthroline copper (I)—known to be an intercalator [47]—has been observed to mimic the micrococcal-nuclease cutting patterns both in vitro as well as in vivo. For these reasons, the existence of premeltons in DNA predicts irehdiamine and ethidium to be competitive-inhibitors of the pancreatic-DNase I and the micrococcal-nuclease cleavage reactions, both in naked DNA and in DNA in active- and inactive-chromatin.

Figure 10 shows the central beta-structural element alternating between its lowest- and highest energy states within the centers of B–B (or A–A) premeltons—“pinned” and “unpinned” by irehdiamine (left) and ethidium (right). These motions reflect the presence of dynamical breather- motions within premeltons, which facilitate the ability of drugs and dyes to intercalate into DNA.

## What is the relationship between DNA breathing and drug-intercalation?

**Fig. 11:**
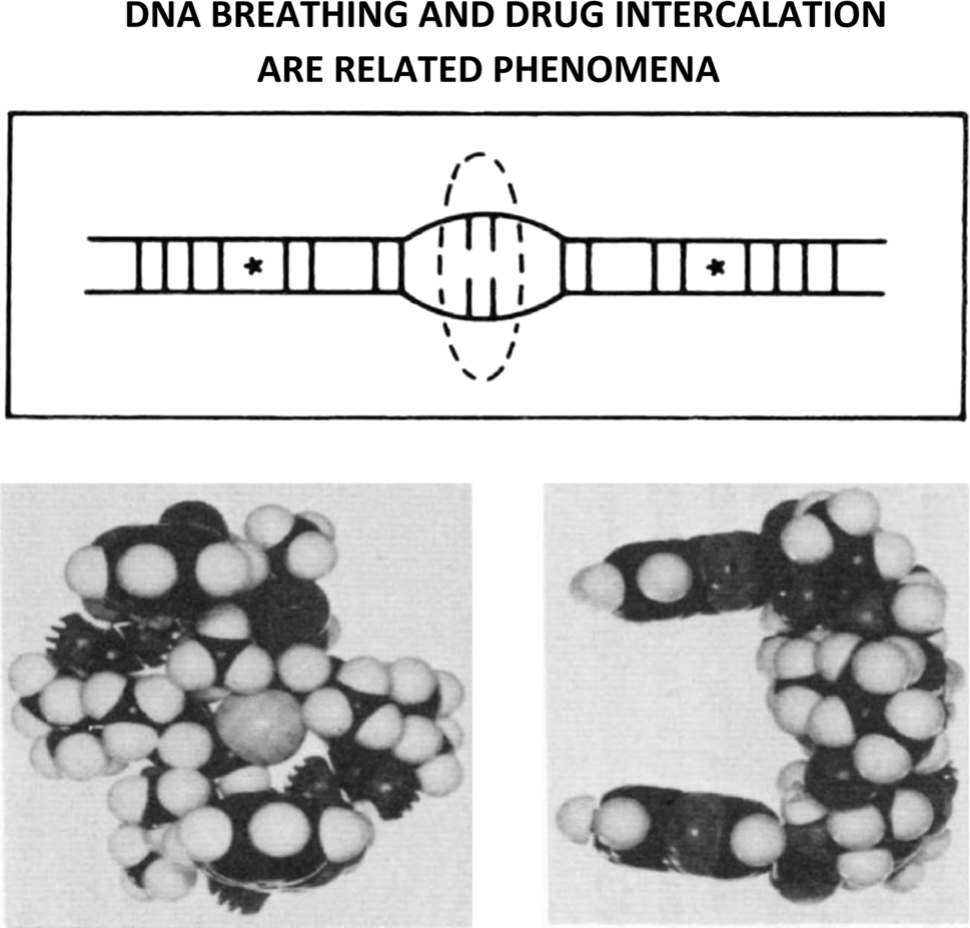
*Top* A simplified illustration to show DNA-breathing, a concerted dynamical process within premeltons that combines base-pair unstacking with the transient-rupture of hydrogen-bonds connecting base-pairs. *Bottom* Echinomycin is an example of a bifunctional-intercalator, having two quinoxaline ring-systems separated by 10.2 Angstroms connected through amide-linkages to a rigid octapeptide-chain. The stereochemistry of this naturally occurring DNA-binding antibiotic necessitates both quinoxaline ring-systems be able to intercalate simultaneously into neighboring high-energy beta-structural elements, and for this reason, is a valuable probe to understand the detailed stereochemistry of DNA-breathing [44, 45]

Figure 11 (top) shows a simplified illustration of DNA-breathing, a concerted dynamical process within premeltons that combines base-pair unstacking with the transient rupture of hydrogen-bonds connecting base-pairs. Premeltons are proposed to arise spontaneously at the early-melting regions of DNA to nucleate DNA-melting, their central (beta-DNA) core-regions serving as activated-intermediates that allow DNA-breathing and the intercalation of drugs and dyes to take place. Base-pairs undergoing H-bond breakage in the higher-energy more centrally-located beta-DNA regions within premeltons have been indicated by the dashed oval-area. Lower-energy beta-structural elements on either side are marked with asterisks. Kink and antikink regions have not been indicated in this figure.

Figure 11 (bottom) shows the bifunctional-intercalator echinomycin, having two quinoxaline ring-systems separated by 10.2 Angstroms connected through amide-linkages to its rigid octapeptide-chain. The stereochemistry of this naturally occurring DNA-binding antibiotic necessitates both quinoxaline ring-systems be able to intercalate simultaneously into neighboring high-energy beta-structural elements and, for this reason, is a valuable probe to understand the detailed stereochemistry of DNA breathing [44, 45].

## How does this porphyrin intercalate into (and out of) DNA?

**Fig. 12:**
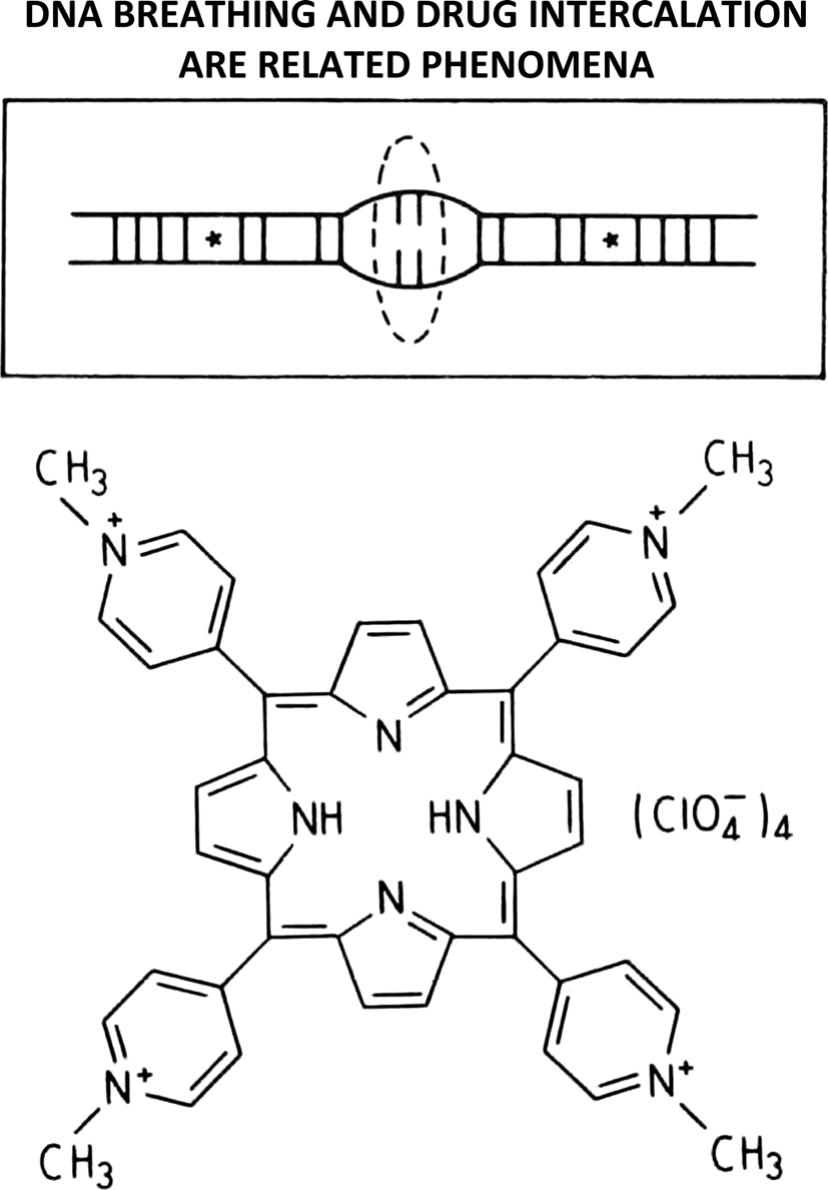
Intercalators that necessitate the transient rupture of hydrogen-bonds connecting base-pairs to gain entrance into (and exit out of) DNA (i.e., meso-tetra [4-N-methyl (pyridyl) porphine]) constitute convincing evidence that DNA-breathing and drug-intercalation are related phenomena [46]

Intercalators that necessitate the transient rupture of hydrogen-bonds connecting base‐pairs to gain entrance into (and exit out of) DNA (i.e., meso-tetra [4-N-methyl (pyridyl) porphine] constitute convincing evidence that DNA-breathing and drug‐intercalation are related phenomena (see Fig. 12) [46].

## How does DNA melt?

**Fig. 13:**
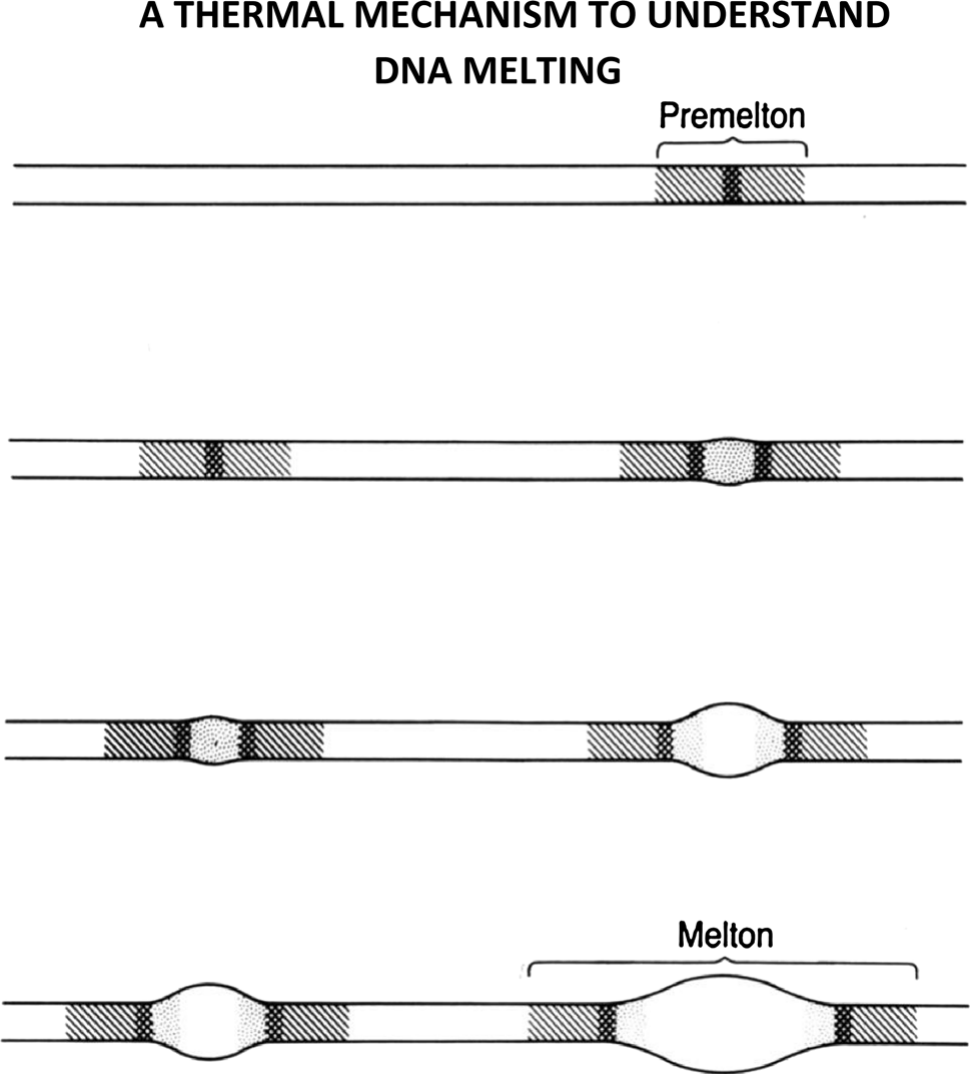
A schematic illustration of DNA-melting showing how premeltons become meltons with increasing temperature—these being examples of structural-solitons in DNA. See text below for further discussion

Figure 13 shows a schematic illustration of DNA melting—showing how premeltons become meltons with increasing temperature—these being examples of structural solitons in DNA.

Premeltons form at the early melting regions in DNA—and at elevated temperatures—serve to nucleate the melting process. At lower temperatures, kink and antikink pairs surround small beta-DNA core regions.

As the temperature rises, these kink-antikink pairs move apart, leaving growing beta-DNA cores, whose inner regions begin to experience the nonlinear stretching of hydrogen-bonds connecting base-pairs.

Finally, at still higher temperatures, these hydrogen bonds break and single-stranded melted regions appear—separated from regions of B- (or A-) DNA by the complex phase-boundaries just described. Such composite-structures correspond to higher-energy structural-solitons, and are called—*meltons*.

## Do premeltons exist at the 5′- and 3′- ends of genes?

**Fig. 14:**
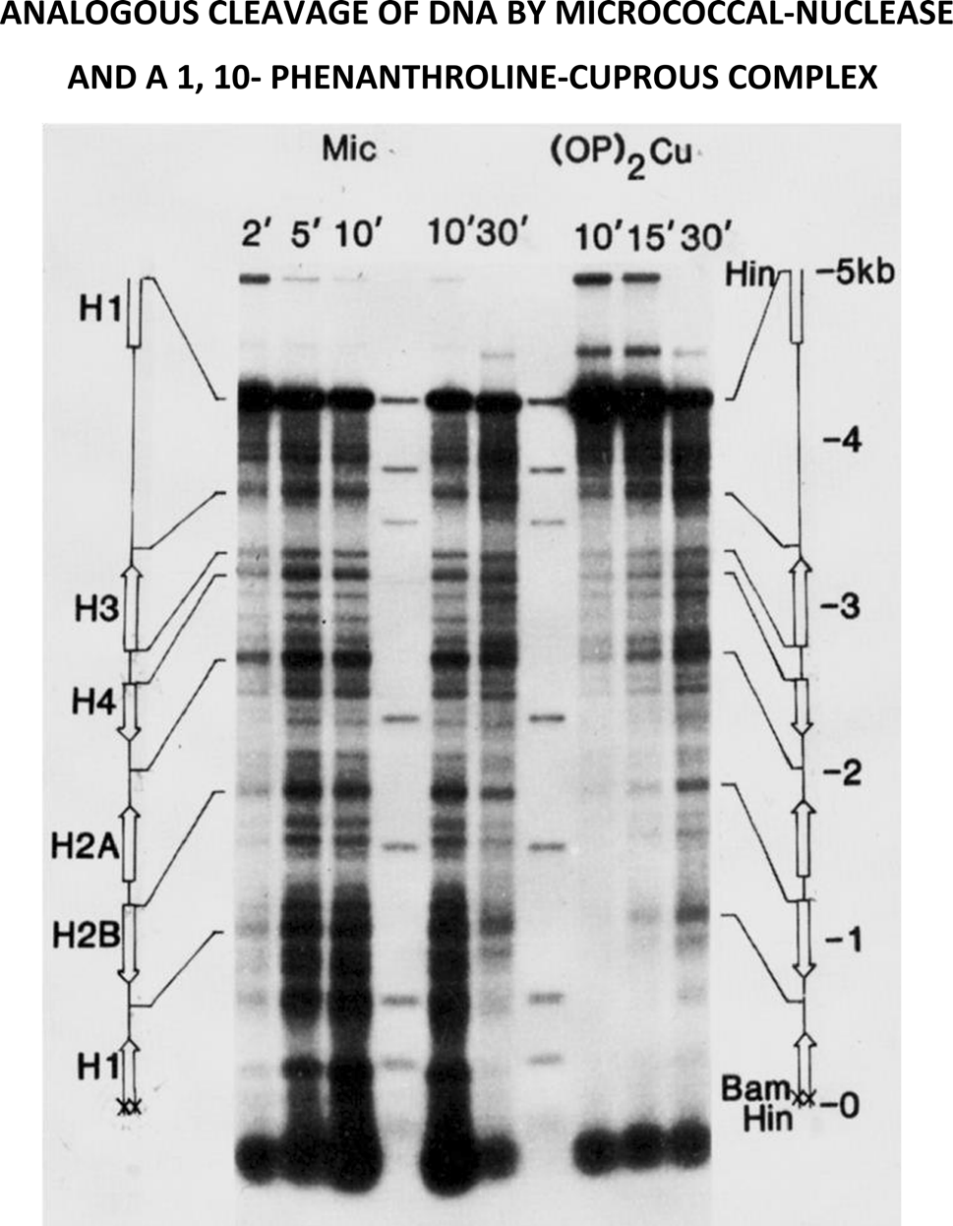
This study demonstrates the remarkable similarities between the chemical-nuclease, 1, 10- phenanthroline copper(I)—a known intercalator [47]—and the micrococcal-nuclease in their ability to recognize and to cleave hypersensitive-sites in a 5000 base‐pair circular‐DNA fragment containing the histone gene-cluster from *D*. *melanogaster* [48]. See text for additional discussion

Figure 14 shows a comparison between the micrococcal-nuclease and 1, 10-phenanthroline-copper (I) cleavage patterns, using agarose-gel electrophoresis, followed by autoradiography [48]. Circularized naked DNA-molecules, previously labeled with radioactive phosphorous at a single Bam H1 site, were incubated with either the micrococcal-nuclease or 1, 10-phenanthroline-copper (I), and the reaction followed as a function of time.

The resulting fragments were then cleaved with Hind III to give fragments having a common Hind III end, this being 68 base-pairs downstream from the labeled Bam site. Slab gel electrophoresis in 1 % agarose, followed by autoradiography, was then used to visualize radioactively-labeled fragments containing different DNA chain-lengths.

Cleavage patterns exhibited by both agents are amazingly similar, most hypersensitive sites being found at the 5′ ends of genes, or lying between adjacent genes. What is even more remarkable is the observation in subsequent experiments, that many of these same sites nucleate DNA-melting, when the single-strand specific DNA-binding protein of *E. coli* is added to these same circular DNA molecules, made negatively superhelical. The location of these small melted regions has been established using the S‐1 nuclease, in combination with electron-microscopy [49].

Important additional information has been provided by studies of this same gene-cluster in active chromatin—where the micrococcal-nuclease cleaves hypersensitive-sites at the 5′ ends of genes, while the pancreatic-DNase cleaves hypersensitive-sites at *both* the 5′ and 3′ ends of genes [50].

Taken together, these data indicate the presence of premeltons at the beginning and ends of genes, playing a key role in determining the initiation and the termination of DNA transcription.

**Fig. 15:**
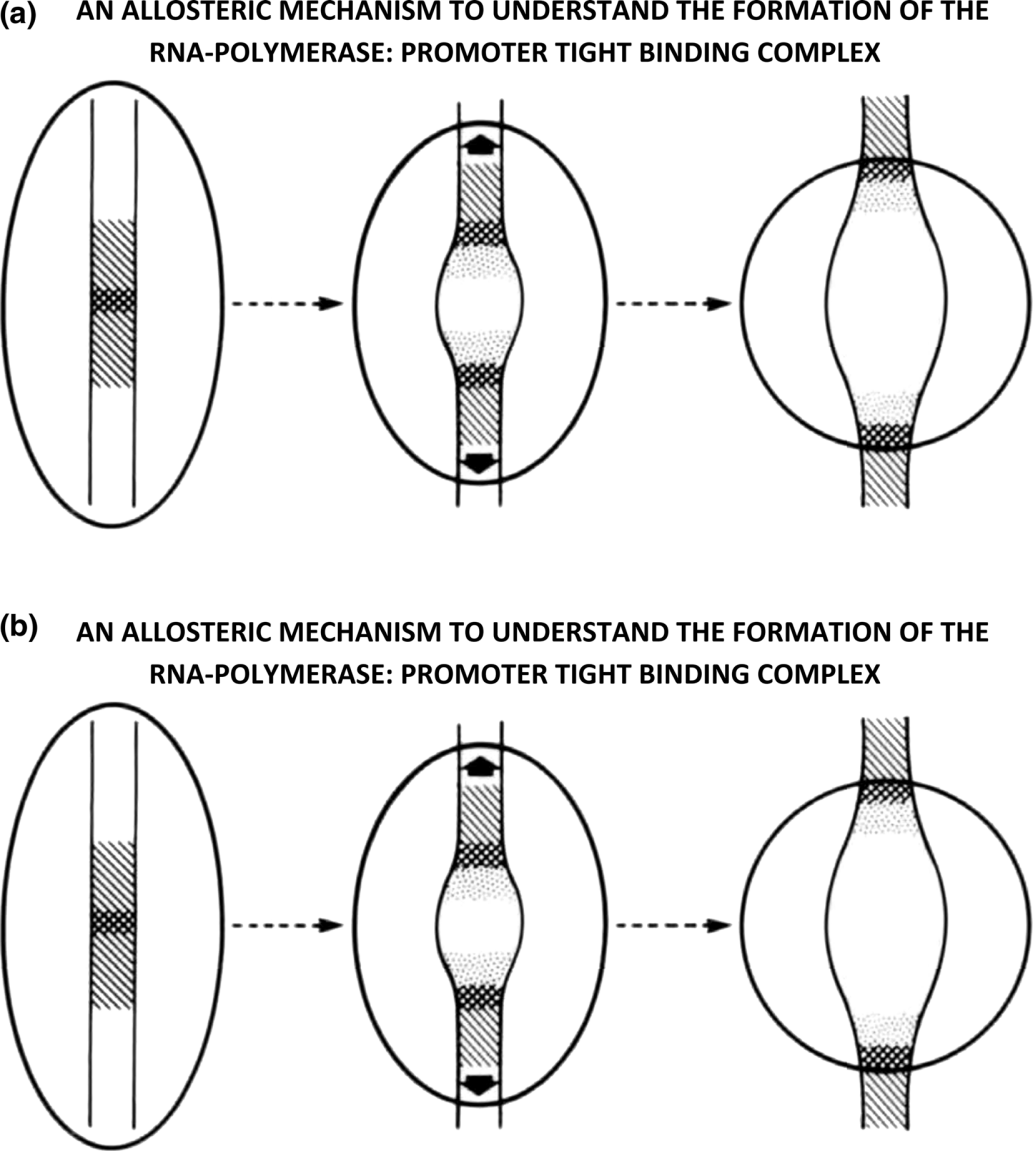
**a** One can envision the formation of the transcriptionally competent tight–binding complex to involve the initial attachment of the polymerase to a premelton located at or near the start of transcription (shown on the *left*), triggering a cascade of conformational changes in both the polymerase and the DNA (shown in the *middle*), that lead to the formation of the tight–binding complex (shown on the *right*). **b** The mechanism is reversible—one can envision the transcriptionally-competent tight–binding complex (shown on the *right*) to undergo a series of concerted allosteric–transitions (shown in the *middle*) that lead to the final-detachment of the polymerase from the premelton (shown on the *left*). Such a mechanism necessarily accompanies the termination of transcription at the 3′ ends of genes. The level of negatively-superhelical strain-energy in DNA provides the bias that determines the direction of this protein-DNA phase-transition. Other more active processes can be involved as well

Their presence immediately suggests an allosteric mechanism that underlies the formation of the RNA polymerase: promoter tight-binding complex (see Fig. 15a, b).

One can envision the formation of the transcriptionally-competent tight-binding complex to involve the initial attachment of the polymerase to a premelton located at or near the start of transcription (shown on the left), triggering a cascade of conformational changes in both the polymerase and the DNA (shown in the middle), that lead to the formation of the tight-binding complex (shown on the right).

The process described above can be considered to be a series of concerted allosteric-transitions leading to the progressive-union of two molecular species. How might this occur, and what is its underlying energetics?

*This is best understood as being a protein-DNA structural phase-transition, the emergent phase being the RNA polymerase: promoter tight-binding complex*. Complex formation entails a series of stepwise conformational transitions, in which energy is transferred from the polymerase to the DNA in the form of small packets (being referred to as an “avalanche of kinks” by physicists). This is possible, provided the protein begins by being in a high-energy metastable-state. It can then spontaneously fall into lower lying metastable-states as DNA-melting and tight complex-formation ensue. Such an adiabatic process is expected to have little (or no) change in free-energy.

The mechanism is reversible—one can imagine the transcriptionally-competent tight-binding complex (shown on the right) to undergo a series of concerted allosteric-transitions (shown in the middle) that lead to the final-detachment of the polymerase from the premelton (shown on the left). Such a mechanism necessarily accompanies the termination of transcription at the 3′ ends of genes. The level of negatively-superhelical strain-energy in DNA provides the bias that determines the direction of this protein-DNA phase-transition. Other more active processes can be involved as well.

It is well known that the transcriptionally-competent tight-binding complex is associated with an extremely large (apparent) binding-constant. Classical thermodynamics would predict a large net negative free-energy change to accompany the binding-reaction. *If this were true, how then is it possible for the RNA-polymerase to move along DNA during the process of DNA-transcription*?

This is understood in the following way. The binding by the RNA-polymerase to the promoter is an adiabatic-process, energy being transferred from the protein to the DNA in a series of stepwise allosteric-transitions that lead to the formation of the transcriptionally-competent tight-binding complex (see above). Although there is little or no net free-energy change expected for such a process (*this being an example of a protein-DNA structural phase-transition*), the final-structure contains both molecular-species topologically linked-together (i.e., in a way analogous to how two oppositely-oriented “easy-zippers” are connected together, when becoming attached to the tracks on a “Ziploc” plastic bag). Such a model predicts the transcription-complex to be able to “slide” with minimal friction along DNA during transcription, in spite of the large apparent binding-constant holding these molecular-species together. This model accounts for the processivity observed in RNA synthesis as well.

The tight-binding transcriptionally competent complex arises as the result of topological-linking (i.e., intertwining)—not from the presence of a large negative free-energy change accompanying complex formation. The extremely large (apparent) binding constant for this complex, therefore should not be confused with a true equilibrium binding constant—as described by classical thermodynamics.

## How does actinomycin bind to DNA and exert its mechanism of action?

**Fig. 16:**
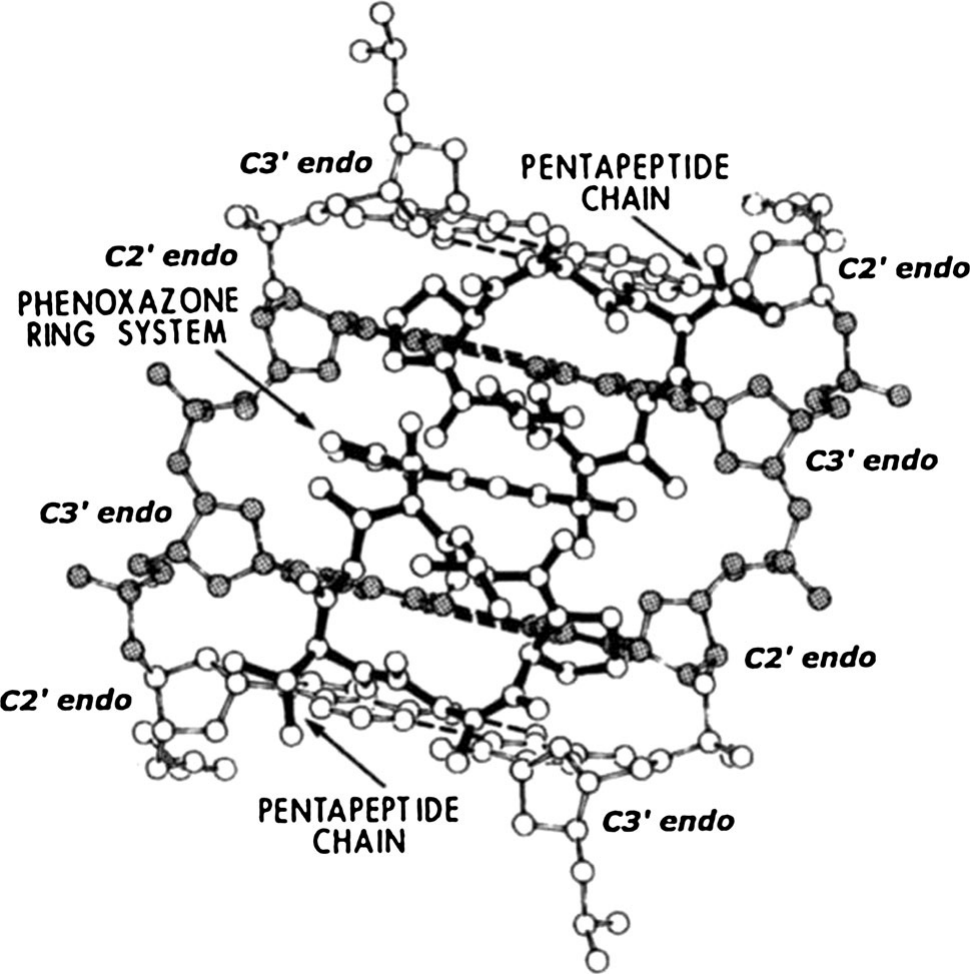
The actinomycin: beta-DNA binding model

It intercalates into its highest-energy beta-DNA form found within the boundaries connecting double-stranded B-DNA with single-stranded DNA in the transcription-complex (see Fig. 16). This immobilizes (i.e., “pins”) the complex, interfering with the elongation of growing RNA-chains.

**Fig. 17:**
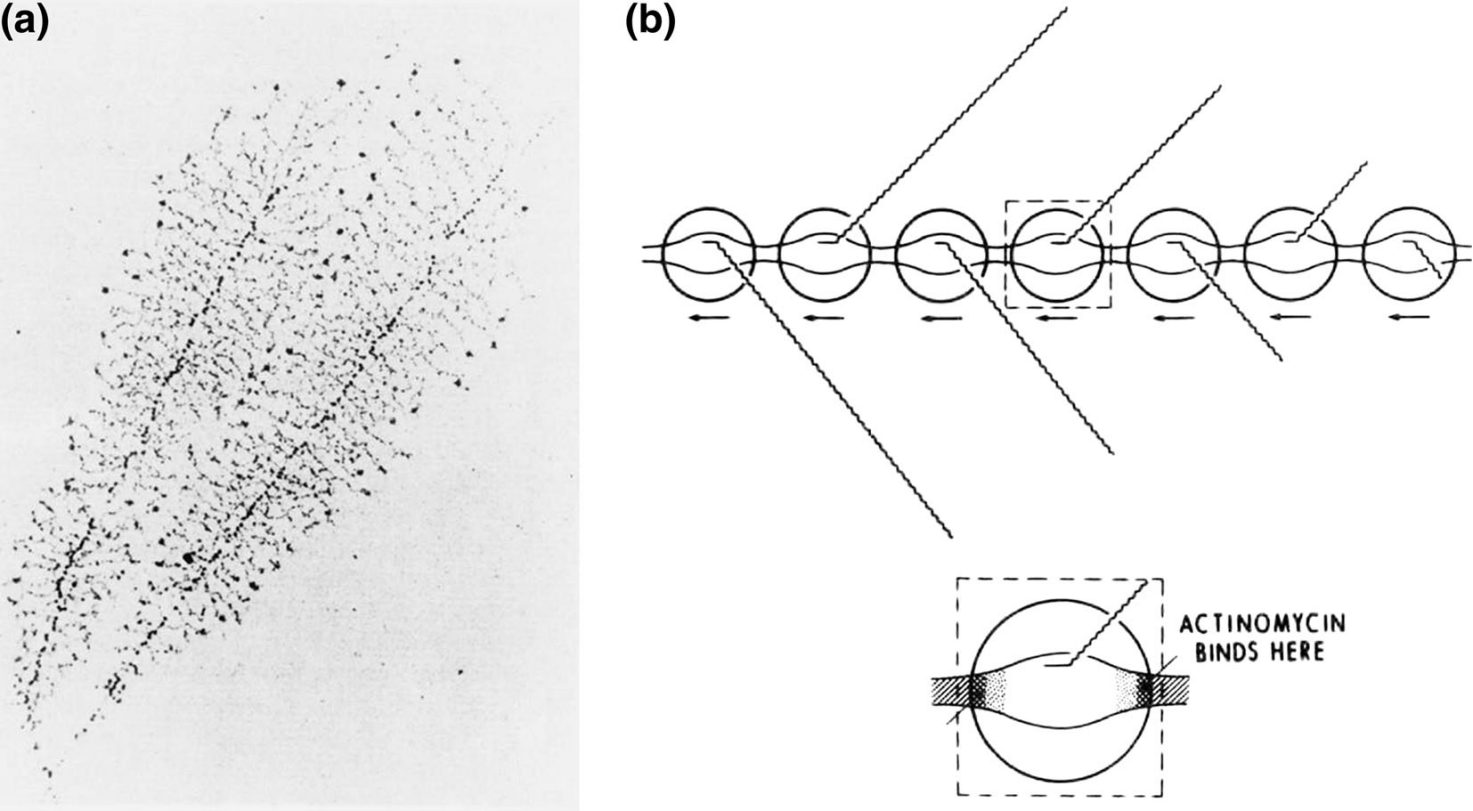
How actinomycin inhibits nucleolar RNA-synthesis

## More precisely—how does actinomycin inhibit nucleolar RNA synthesis?

See Fig. 17a, b.

Leroy Liu and James Wang have provided a key insight into the nature of DNA supercoiling accompanying transcription that has shed additional light on this question [51]. They have theorized that—in the presence of significant resistance to the rotational motion of the RNA polymerase and its nascent RNA chain around DNA during transcription—the advancing polymerase generates positive superhelicity in the DNA template ahead of it, and negative superhelicity behind it.

In nucleolar genes, where there may be as many as 200 RNA polymerases moving down the DNA template while synthesizing growing ribosomal RNA-chains [52]—positive and negative superhelical DNA regions between them annihilate one-another, causing adjacent transcription-complexes to bond-together to form “trains” of transcription-complexes, these now moving synchronously along DNA. *If this were the case, then the binding by one actinomycin molecule is sufficient to stop the entire “transcription-train” from moving along DNA*.